# Data from a targeted proteomics approach to discover biomarkers in saliva for the clinical diagnosis of periodontitis

**DOI:** 10.1016/j.dib.2018.03.036

**Published:** 2018-03-12

**Authors:** V. Orti, B. Mertens, J. Vialaret, P. Gibert, A. Relaño-Ginés, S. Lehmann, D. Deville de Périère, C. Hirtz

**Affiliations:** a U.F.R. d’Odontologie, Département de Parodontologie, 545 avenue du Professeur Jean-Louis Viala, 34193 Montpellier Cedex 5, France; b University of Montpellier, LBPC- IRMB, CHU de Montpellier, 80 rue Augustin Fliche, Montpellier, France

**Keywords:** Clinical chemistry, Mass spectrometry, Proteomics, Saliva biochemistry, Oral disease, Periodontitis

## Abstract

This study focused on the search for new biomarkers based on liquid chromatography-multiple reaction monitoring (LC-MRM) proteomics profiling of whole saliva from patients with periodontitis compared to healthy subjects. The LC-MRM profiling approach is a new and innovative method that has already been validated for the absolute and multiplexed quantification of biomarkers in several diseases. The dataset for this study was produced using LC-MRM to monitor protein levels in a multiplex assay, it provides clinical information on salivary biomarkers of periodontitis. The data presented here is an extension of our recently published research article (Mertens et al., 2017) [1].

## Graphical abstract

**Figure:**
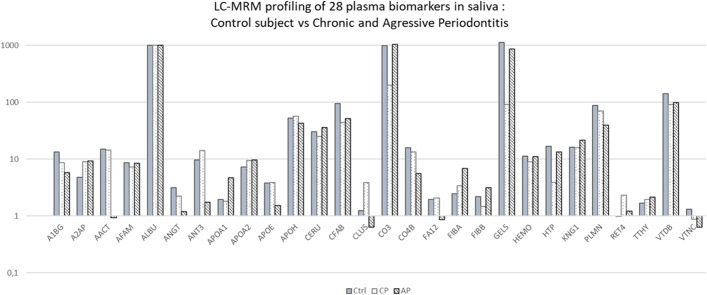

**Specifications Table**

**Table:** 

Subject area	*Biology*
More specific subject area	*Clinical chemistry, Salivary biomarkers, Oral disease, Periodontitis*
Type of data	*Tables, figures*
How data was acquired	Liquid chromatography (LC) separation was carried out on a 1290 LC system (Agilent Technologies). Peptides were resolved on a reverse-phase column over 30 min at a flow-rate of 400 μL/min.
	Peptides were then analyzed on a triple quadrupole mass spectrometry (MS) system (6490, Agilent Technologies), equipped with an Agilent Jet Stream ESI interface. The MS was operated in dynamic MRM mode with a retention time window of 2.5 min and a maximum cycle time set at 700 ms.
Data format	*Analyzed and processed data*
Experimental factors	*Whole saliva samples were collected without stimulating salivation.*
	*Samples, with a minimum volume of 3 mL of saliva, were collected over 5 min from each patient and healthy subject using cotton swabs (Sarstedt Salivette®), placed in the mouth between the upper jaw and the parotid duct close to the first molar. Samples from 33 patients were immediately centrifuged for 15 min at 2600×g, 4 °C to remove residual particles, such as bacteria and mucosal cells. After centrifugation, the supernatant was aliquoted in 1.5 mL Eppendorf tubes and stored at − 80 °C until analysis.*
Experimental features	*Salivary protein concentrations were determined using a colorimetric protein assay (Bicinchoninic Acid Protein Assay Kit; BCA) and a fixed amount (70 µg total protein) was used. Sample preparation was performed in triplicate. Proteins were denatured/reduced/alkylated/digested with trypsin on an AssayMAP Bravo Platform (Agilent). The peptides generated were cleaned on C18 tips before spiking with a mixture of 35 Stable Isotope Labeled Standard peptides. Samples were then injected in duplicate into the liquid chromatography-mass spectrometry (LCMS) system. In total, 28 proteins were quantified and their levels compared between three patient groups (Control, Aggressive Periodontitis and Chronic Periodontitis).*
Data source location	*Institute for Regenerative Medicine & Biotherapy, Montpellier Hospital, France*
Data accessibility	*Data presented in article*

**Value of the data**

• These data present a comprehensive view of plasmatic biomarkers screened in saliva in a context of Periodontitis.
• Quantitative values from the study cohort establish the clinical range in saliva for the 28 biomarkers presented in the article.
• These LC-MRM data should be very helpful for researchers dealing with clinical issues related to the diagnosis of periodontal diseases.

## 1 Data

In this Data in Brief article, we present the salivary profiling of 28 plasma biomarkers from 33 patients (12 healthy individuals (control), 10 Chronic Periodontitis patients (CP) and 11 patients with Aggressive Periodontitis (AP)). Subjects were between 18 and 75 years of age. Saliva samples were processed in triplicate and analyzed in duplicate by LC-MRM. Quantitative results obtained for the 28 protein biomarkers studied were validated and are presented together in Table 1. Absolute quantification was obtained by spiking labeled proteotypic peptides containing heavy isotope equivalents of arginine ([13C6] or [13C6, 15N4]) or lysine ([13C6] or [13C6, 15N2]) residues. The analytical performance obtained for each biomarker is indicated in Table 1, based on the following criteria: Linear concentration range (pg/mL), Linear response (R2), Limit of detection (LOD, pg/mL), Limit of quantification (LOQ, pg/mL), precision (CV %), clinical range in saliva samples (pg/mL).

**Table 1:** Analytical performances and clinical results on the multiplex proteins.

**Quantified protein in saliva**	**Calibration curve concentration range (µg/mL)**	**Linear response (R**^**2**^**)**	**LOD (µg/mL)**	**LOQ (µg/mL)**	**Clinical range on saliva samples (µg/mL)**	**Precision on all the patient samples (%)**
Afamin (AFAM)	0.198–39.535	0.9825	0.269	0.799	< 0.269–19.472	20.6
Alpha-1-antichymotrypsin (AACT)	0.134–26.880	0.9951	0.156	0.242	0.167–9.441	16.9
Alpha-1B-glycoprotein (A1BG)	0.031–6.166	0.9923	0.052	0.061	0.083–8.426	13.6
Alpha-2-antiplasmin (A2AP)	0.149–11.983	0.9529	0.376	1.200	< 0.376–4.519	17.2
Angiotensinogen (ANGT)	0.029–5.909	0.9970	0.043	0.097	< 0.043–3.197	24.2
Anti-thrombin-III (ANT3)	0.058–3.329	0.9951	0.069	0.176	0.089–1.577	21.0
Apolipoprotein A-I (ApoA1)	0.083–1.667	0.9508	0.073	0.095	< 0.073–2.220	9.7
Apolipoprotein A-II (ApoA2)	0.258–10.342	0.9181	0.395	0.48	< 0.395–5.781	9.0
Apolipoprotein E (ApoE)	0.102–8.132	0.9761	0.156	0.165	0.157–4.327	9.4
Beta-2-glycoprotein I (ApoH)	1.076–43.058	0.9704	0.690	1.098	0.749–47.833	14.2
Ceruloplasmin (CERU)	0.071–7.131	0.9676	0.021	0.290	0.075–25.019	25.7
Clusterin (CLUS)	0.014–1.189	0.9883	0.015	0.030	0.021–1.141	19.8
Coagulation factor XII (FA12)	0.118–2.354	0.9929	0.053	0.060	< 0.053–2.195	12.4
Complement C3 (CO3)	1.098–109.828	0.9891	0.002	1.080	0.806–317.369	22.0
Complement C4-B (CO4B)	0.213–8.513	0.9765	0.306	0.336	0.327–7.961	16.9
Complement factor B (CFAB)	0.246–49.288	0.9889	0.004	0.167	0.192–37.879	26.3
Fibrinogen alpha chain (FIBA)	0.271–5.425	0.9303	0.109	0.145	0.114–2.589	9.3
Fibrinogen beta chain (FIBB)	0.151–6.029	0.9938	0.100	0.359	< 0.100–4.889	25.9
Gelsolin (GELS)	0.493–49.263	0.9859	0.001	0.087	0.425–518.277	17.8
Haptoglobin (HPT)	0.129–10.297	0.9826	0.153	0.156	< 0.153–23.009	17.2
Hemopexin (HEMO)	0.029–5.855	0.9939	0.053	0.054	0.058–12.232	15.4
Kininogen-1 (KNG1)	0.042–8.301	0.9956	0.111	0.125	0.251–18.172	16.7
Plasminogen (PLMN)	0.525–21.005	0.9845	0.388	0.548	0.652–22.216	23.8
Retinol-binding protein 4 (RET4)	0.006–1.251	0.9842	0.034	0.103	< 0.034–1.645	17.1
Serum Albumin (ALBU)	0.789–39.473	0.9834	0.034	3.263	9.135–602.306	19.1
Transthyretin (TTHY)	0.008–1.6343	0.9905	0.021	0.027	0.037–2.569	17.0
Vitamin D binding protein (VTDB)	0.304–30.429	0.9941	0.001	0.224	0.659–77.176	19.6
Vitronectin (VTNC)	0.031–1.242	0.9933	0.014	0.042	< 0.014–1.005	19.3

**Fig. 1:**
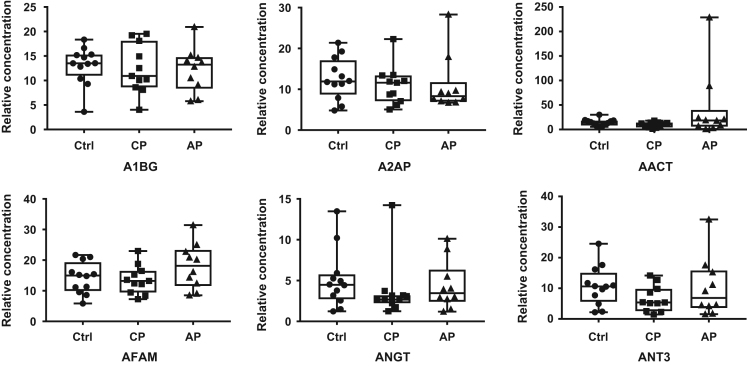
Graphic results of normalized protein levels for the protein multiplex. All the proteins were presented as medians and interquartile ranges. Statistical analysis (Mann-Whitney test) was used to evaluate the significance of the difference between the groups.

The three disease status groups were compared after normalization for albumin concentration to adjust for analytical variability (Fig. 1).

The significance of any difference between the groups was statistically analyzed (Mann-Whitney test). Four proteins (Beta-2-glycoprotein I (APOH); α-fibrinogen (FIBA); Hemopexin (HEMO); Plasminogen (PLMN)) were found to be present at statistically different levels between groups, and are promising candidates to facilitate screening and diagnosis of periodontal diseases.

## 2 Experimental design, materials and methods

Experimental design and the materials and methods have been reported previously [1].

### 2.1 Patient selection/population

Patients (*n* = 33) were recruited and screened at the Department of Periodontology, CHRU Montpellier (France): 10 were diagnosed with CP, 11 with AP and 12 were healthy controls. The control group was composed of patients presenting no evidence of periodontitis. Subjects’ ages were between 18 and 75 years. Smoking status (current, former, never) was recorded for all individuals, with heavy smoking (> 15 cig/d) used as an exclusion criterion.

Patients gave informed consent for participation in the study, and for conservation of their samples in a registered, ethically-approved biological collection (#DC-2008-417) that is stored at Montpellier University Teaching Hospital's certified NFS 96-900 biobank (Ref: BB-0033-00031 www.biobanques.eu). Authorization to handle personal data was granted by the French Data Protection Authority (CNIL) under number 1709743 v0.

### 2.2 Sample collection and preparation

Whole saliva samples were collected without stimulating salivation. At the time of collection, subjects had fasted for 60 min, had not smoked in the preceding 4 h and had abstained from drinking alcohol in the previous 12 h. Patients were asked to rinse their mouths and to drink a glass of water prior to sample collection. Samples – minimum volume 3 mL of saliva – were collected over 5 min from each patient and healthy subject using cotton swabs (Sarstedt Salivette®, Nümbrecht, Germany), which were placed in the mouth between the upper jaw and the parotid duct, close to the first molar. Samples were then immediately centrifuged for 15 min at 2600*g*, 4 °C to remove residual particles, such as bacteria and mucosal cells. Colorimetric protein assay (BCA Protein Assay Kit, Thermo Scientific Pierce, USA) with bovine serum albumin (BSA) as standard was used to quantify salivary protein concentrations. Salivary samples were also visually inspected to detect any pink coloration that could be due to contamination with blood. Samples that were not perfectly clear were discarded. After centrifugation, the supernatant was aliquoted in 1.5 mL Eppendorf tubes and stored at − 80 °C until analysis.

### 2.3 Sample preparation

To correct inter-individual differences in protein concentration, a fixed amount of total protein (70 µg) was used for analysis. After a short centrifugation of the sample (500 g, 2 min, room temperature), it was mixed with 200 µL cold ethanol before placing it at − 20 °C overnight. After centrifugation (17,000*g*, 5 min, 4 °C), the supernatant was discarded and the protein pellet was resuspended in 20 µL 8 M Urea under agitation (1500 rpm) for 10 min.

Samples were then transferred to 96-well plates and processed. Briefly, reduction, alkylation, digestion and clean-up were performed using an automated AssayMAP Bravo Platform (Agilent, Lexington, USA). Digestion was allowed to proceed overnight at 37 °C and samples were then dried using a vacuum concentrator (Labconco, Kansas, USA). Before analysis, samples were resuspended in 20 µL of 2% acetonitrile/0.1% formic acid/97.9% water and spiked with the heavy peptide mix (1/100) described below. Samples were agitated for 10 min. All samples were prepared in triplicate and duplicate analyses were performed.

### 2.4 LC-MRM method

Peptides were separated using a 1290 LC system (Agilent Technologies) and were analyzed on a triple quadrupole mass spectrometry (MS) system (6490, Agilent Technologies), equipped with an Agilent Jet Stream ESI interface and working in positive ion mode.

MS was operated in dynamic MRM mode with a retention time window of 2.5 min and a maximum cycle time set at 700 ms. One peptide per protein and three transitions per peptide were monitored. As both light and heavy versions of each peptide were present in samples, a total of six transitions were monitored per protein.

Precursor ions were selected in the first quadrupole, ion funnel RF high pressure was set to 150 V and low pressure to 60 V. Ion transfer was performed at a fragmentor voltage of 380 V and a cell accelerator voltage of 5 V. Collision energies were optimized for each transition.

Labeled standard peptides and generation of the calibration curve:

A commercial kit for the absolute quantification of a 35-protein panel is available with one labeled proteotypic peptide per protein (PeptiQuant™ Performance Kit, Victoria, Canada). Labeled peptides contained heavy isotope forms of arginine ([13C6] or [13C6, 15N4]) or lysine ([13C6] or [13C6, 15N2]) amino acid residues (Cambridge Isotope Laboratories, Andover, MA, USA) at their C-terminus. The following proteins were monitored: Afamin (AFAM), Alpha-1-antichymotrypsin (AACT), Alpha-1B-glycoprotein (A1BG), Alpha-2-antiplasmin (A2AP), Angiotensinogen (ANGT), Anti-thrombin-III (ANT3), Apolipoprotein A-I (ApoA1), Apolipoprotein A-II (ApoA2), Apolipoprotein E (ApoE), Beta-2-glycoprotein I (ApoH), Ceruloplasmin (CERU), Clusterin (CLUS), Coagulation factor XII (FA12), Complement C3 (CO3), Complement C4-B (CO4B), Complement factor B (CFAB), Fibrinogen alpha chain (FIBA), Fibrinogen beta chain (FIBB), Gelsolin (GELS), Haptoglobin (HPT), Hemopexin (HEMO), Kininogen-1 (KNG1), Plasminogen (PLMN), Retinol-binding protein 4 (RET4), Serum Albumin (ALBU), Transthyretin (TTHY) and Vitamin D binding protein (VTDB).

A calibration curve was produced by adding heavy standards to a real matrix of pooled saliva samples. After in-vial dilution, the pool of heavy peptides was mixed with the matrix. Spiked samples were analyzed by LC–MS. The range for the calibration curve covered two orders of magnitude over eight points (1/10; 1/25; 1/50; 1/100; 1/200; 1/500; 1/1000; 1/2000), and points were analyzed in triplicate. The calibration curve was freshly prepared daily.

### 2.5 Data analysis

Skyline® 3.6 open source software (https://skyline.ms/project/home) was used to analyze quantitative data and generate calibration curves. Peak detection was performed automatically by the software and checked manually. Excel software was used to generate calibration curves and to quantify proteins in the samples.

### 2.6 Statistical analysis

Statistical analyses were performed using GraphPad Prism 7.03. A Mann-Whitney test was used to compare categorical variables between groups. The significance threshold was set to *p* < 0.05.
