# Supplementary material for: Data from a targeted proteomics approach to discover biomarkers in saliva for the clinical diagnosis of periodontitis
Source: Data Brief. 2018 Mar 12;18:294–9. doi: 10.1016/j.dib.2018.03.036 (PMC5996262; doi:10.1016/j.dib.2018.03.036)
Supplement: Supplementary file 1 — Transparency document [file mmc1.doc]

**Conflict of Interest Form**

**Data In Brief**

Corresponding author: Christophe Hirtz ([christophe.hirtz@umontpellier.fr](mailto:christophe.hirtz@umontpellier.fr))

University of Montpellier, CHU Montpellier, Institute of Regenerative Medicine & Biotherapy, 80 Avenue Auguste Fliche, Montpellier, 34925, France. Electronic address: [christophe.hirtz@umontpellier.fr](mailto:christophe.hirtz@umontpellier.fr)

Submission date : 01/12/2017

Title: Innovative and multiplexed targeted proteomics approach for the clinical diagnosis of periodontitis using saliva samples

Authors: B. Mertens^1^, V. Orti^1^ J. Vialaret^2^, P. Gibert^1^, A Relaño-Ginés^2^, S Lehmann^2^ , D. Deville de

Périère^2^and C. Hirtz^2^

Affiliations:

1 - U.F.R. d’Odontologie, Département de Parodontologie, 545, avenue du Professeur Jean-Louis Viala, 34 193 Montpellier Cedex 5

2 – University of Montpellier, LBPC- IRMB, CHU de Montpellier, 80 rue Augustin Fliche, Montpellier, France

Contact email: [christophe.hirtz@umontpellier.fr](mailto:christophe.hirtz@umontpellier.fr)

I certify that this research is original, not under publication consideration elsewhere, and free of conflict of interest.

**Authorship**

I certify that I am the corresponding author for this manuscript. The manuscript is submitted with the knowledge and on behalf of the listed co-authors.

I certify that each co-author listed above participated sufficiently in the work to take responsibility for the content, and that all those who qualify are listed. Contributors

I certify that no funded writing assistance was utilised in the production of this manuscript.

**Author disclosures**

I certify that none of the authors have any relevant financial and/or nonfinancial relationships to disclose.
